# Trace-Level Determination of Polycyclic Aromatic Hydrocarbons in Dairy Products Available in Spanish Supermarkets by Semi-Automated Solid-Phase Extraction and Gas Chromatography–Mass Spectrometry Detection

**DOI:** 10.3390/foods11050713

**Published:** 2022-02-28

**Authors:** Laura Palacios Colón, Andrés J. Rascón, Evaristo Ballesteros

**Affiliations:** Department of Physical and Analytical Chemistry, E.P.S of Linares, University of Jaén, 23700 Linares, Spain; lpcolon@ujaen.es (L.P.C.); arascon@ujaen.es (A.J.R.)

**Keywords:** dairy product composition, priority pollutants, polycyclic aromatic hydrocarbons, health risk, liquid–liquid extraction, continuous solid-phase extraction, gas chromatography-mass spectrometry

## Abstract

Polycyclic aromatic hydrocarbons (PAHs) have been classified as priority pollutants by the U.S. Environmental Protection Agency (EPA) and the European Commission on the grounds of their carcinogenic, mutagenic and teratogenic properties. Because of their ubiquity in industrial processes and the environment, PAHs can reach milk and dairy products and, eventually, humans. In this work, a new method was developed to detect and quantify sixteen of the EPA’s priority PAHs in commercial milk and dairy products. The method involves liquid–liquid extraction (LLE) followed by semi-automated solid-phase extraction (SPE) to clean up and preconcentrate the analytes prior their detection and quantification by gas chromatography–mass spectrometry (GC–MS). The proposed method provided high precision (relative standard deviation < 11.5%), recoveries of 80–107% and low detection limits (1–200 ng/kg). The method was applied to analyze 30 dairy products, the majority of which contained some PAH at concentrations from 7.1 to 1900 ng/kg. The most-detected analytes were the lighter PAHs (naphthalene, acenaphthylene, fluorene and phenanthrene). None of the samples, however, contained more than four PAHs.

## 1. Introduction

Milk and dairy products are among the most nutritionally complete foods available on the market and have historically been essential to the human diet because of their contents of micro- and macronutrients. This has raised increasing concern with their safety [1], which can be compromised by physical, chemical and microbiological contamination during the animal production of milk or, subsequently, during the transport, storage, processing or delivery of milk and dairy products [2]. Especially prominent among the chemical contaminants are polycyclic aromatic hydrocarbons (PAHs). These are ubiquitous environmental pollutants that are formed by the incomplete combustion of organic matter that can reach food through fuel combustion, industrial processes, degasification, petroleum derivative tasks and also through food processing operations, such as drying, smoking or cooking [3]. While low-molecular-weight PAHs can have systemic effects on targets such as the kidney, blood and liver, their high-molecular-weight counterparts are typically carcinogenic or genotoxic [4]. PAH exposure can lead to health effects in the short and long term. In patients with asthma, PAHs can cause pulmonary impairment and thrombosis. In the long term, PAHs can cause different types of cancer (digestive tract, skin and lungs) [5].

PAHs are non-polar compounds whose lipophilicity makes them soluble in lipids, leading to bioaccumulation in foods and in fat tissue from living organisms [6]. PAHs absorbed by inhalation, ingestion or—in small amounts—through the skin are metabolized by cytochrome-450 enzymes to diol-epoxyde derivatives. The binding of these metabolites to glucuronic acid or sulphates makes them water-soluble and facilitates their excretion in urine, bile or milk [7]. Although human urine is the most widely studied matrix as a biomarker for exposure to PAHs, some of them, such as phenanthrene (Phe), pyrene (Pyr) and benzo(a)pyrene (BaP), are preferentially released through fatty solutions, such as milk. Some studies have shown the need to keep the transfer of PAHs to milk under control to avoid their deleterious effects on food safety [8]. This concern has led several authors to quantify them in milk and dairy products [9]. Thus, Chung et al. (2010) found milk samples from Taiwan containing PAHs at concentrations over the range of 4000 to 310,000 ng/L [10]. Moreover, Lee et al. (2015) found PAHs at levels from 90 to 340 ng/kg in diverse dairy products from South Korea, with benzo(k)fluoranthene (BkF) present at the highest concentrations [11]. Finally, Fasano et al. (2016) determined the concentration and distribution of 10 PAHs in several plant and animal samples of different origins and found various smoked cheeses from Spain containing PAH’s at levels over the range of 20–77,000 ng/kg [12].

Regulation (EU) No 835/2011 established a maximum allowed level of 1 μg/kg for both BaP and the sum of BaP, benzo(a)anthracene (BaA), benzo(b)fluoranthene (BbF) and chrysene (Chry) (also known as PAH4) in infant formula and follow-on milk [13]. Moreover, the U.S. Environmental Protection Agency (EPA) has deemed the sixteen PAHs priority pollutants [14]. In addition, the International Agency for Research on Cancer (IARC) has classified PAHs according to carcinogenicity into three groups: (1) definitely carcinogenic (BaP); (2A) probably carcinogenic (dibenzo[a,h]anthracene and DBahA) and (2B) possibly carcinogenic to humans (BaA, Chry, naphthalene (Nap), indeno[1,2,3-cd]pyrene (IP), BkF and BbF) [15]. A need has, therefore, arisen for accurate, sensitive methods to measure these toxins in foods. Extracting PAHs from milk and dairy products for their determination is a tricky procedure, owing to the high complexity of matrices containing variable amounts of lipids and proteins. Moreover, PAHs are lipophilic and can, thus, easily accumulate in foods (especially in those with high fat contents), which usually require several extraction and cleaning steps before the analytes can be accurately determined [9].

The specific technique to be used to extract PAHs from a food matrix is critically dependent on the nature of the sample. Thus, a cleanup procedure is usually required to remove the coextracted components that potentially interfere with the determination of PAHs in order to avoid matrix effects [9]. Table 1 gathers the recently reported methods for determining PAHs in dairy products. As can be seen, many use a combination of saponification with KOH in methanol or ethanol and liquid–liquid extraction (LLE) with an organic solvent (usually *n*-hexane or cyclohexane) or a solvent mixture (e.g., ethanol/*n*-hexane), followed by cleaning up the extract by solid-phase extraction (SPE) with an appropriate sorbent, such as RP-C18 or silica gel [11,12,16,17,18]. Some methods, however, omit one of the previous steps [10,19,20]. Moreover, some authors have used alternative techniques including solid-phase microextraction [21], in-tube solid-phase microextraction [22], direct immersion solid-phase microextraction [23], ionic liquid liquid-phase micro-extraction [24], Soxhlet plus gel permeation chromatography [25], magnetic solid-phase extraction [20] and QuEChERS [26] to prepare dairy samples.

Gas chromatography (GC) and high-performance liquid chromatography (HPLC) are the two main instrumental choices for the identification and quantification of PAHs [2,9]. Most of the methods in Table 1 use GC coupled to single-quadrupole mass spectrometry (GC–MS) [10,11,19,21,23,27,28] or triple-quadrupole mass spectrometry [25,26], although GC with flame ionization detection is also frequently used instead [27]. On the other hand, photodiode array [24], fluorescence [7,12,16,18,20,29,30,31,32] and mass spectrometry detectors [17] are the main choices for determining PAHs by HPLC. Because some PAHs are not fluorescent, mass spectrometers usually provide more accurate and robust results than fluorescence detectors [3].

The aim of this work is the development of an analytical methodology to detect the EPA’s 16 PAH priority pollutants in milk and dairy products. First, continuous solid-phase extraction (SPE) was used in combination with liquid–liquid extraction (LLE) for the extraction and cleanup of the analytes. Then, the operational variables potentially influencing the performance in the analysis of milk and dairy products were optimized with to the goal of avoiding matrix effects and maximizing sensitivity, selectivity, precision and accuracy. The variables influencing the determination of the analytes by GC coupled to MS in the electron impact (EI) mode were also optimized. The ensuing method was used to quantify PAHs in different types of milk (cow’s, goat’s and sheep’s whole, semi-skimmed and skimmed milk) and dairy products (yogurt, milkshakes, cream, custard, cheese, butter and margarine).

**Table 1:** Selected studies for the presence of PAHs in milk and dairy products.

Samples	Sample Treatment ^a^	Technique ^a^	Analytical Characteristics ^a^	Analytes ^a^	Concentration Found in Real Samples ^b^	References
Milk	LLE + SPE	GC–MS	R: 80–120%	16 PAHs	Nap: 67,000–310,000 ng/L; Ap: 5000–12,000 ng/L; Ac: 58,000–98,000 ng/L; F: 5000–24,000 ng/L; Phe: 21,000–86,000 ng/L; Ant: 18,000 ng/L; Flu: 6000–13,000 ng/L; Pyr: 5000–135,000 ng/L; BaA: 8000–19,000 ng/L; Chry: 19,000–34,000 ng/L; BbF: 6000 ng/L; BkF: 4000 ng/L; BaP: 7000–9000 ng/L	[10]
Milk and dairy product	Saponification + LLE + SPE	GC–MS	LOD: 40–200 ng/kg RSD: 6.8–13.3% R: 87–103%	8 PAHs	Cheese: BaA: 280 ng/kg, BkF: 90 ng/kg, BbF: 340 ng/kg, BaP: 240 ng/kg, BP: 220 ng/kg, DBahA: 210 ng/kg	[11]
Cheese	Saponification + LLE + SPE	HPLC–FLD	LOD: 250–1500 ng/kg RSD: 3.7–7.4% R:66–107%	10 PAHs	Flu: 17,000–77,000 ng/kg; Phe: 44,000–47,000 ng/kg; BaA: 30–14,000 ng/kg; Chry: 30–8800 ng/kg; BbF: 1700–2900 ng/kg; BkF: 20–1600 ng/kg; BaP: 40–5400 ng/kg; B(ghi)P: 90–100 ng/kg; B(ghi)P: 1600–4200 ng/kg; IP: 1600–2800 ng/kg	[12]
Cheese	Saponification + LLE + SPE	HPLC–FLD	LOD: 40–90 ng/kg RSD: 6.5–12.5% R: 73–93%	9 PAHs	Nap: 240–7980 ng/kg; Ac: 100–3060 ng/kg; Ant: 210–820 ng/kg; Pyr: 90–1130 ng/kg; BaA: 80–90 ng/kg; BkF: 60–380 ng/kg; BaP: 60–690 ng/kg; DBahA: 60–730 ng/kg; BP: 70–270 ng/kg	[16]
Heat-treated milk	Saponification + LLE + SPE	HPLC–FLD HPLC–MS	LOQ: 12–201 ng/kg RSD: 0.8−10.4% R: 89–94%	8 PAHs	Phe:1425–1831 ng/kg; Ant:1296–2473 ng/kg; Pyr: 1351–2132 ng/kg; BaA: 813–1072 ng/kg; Chry: 104–261 ng/kg; BkF: 67 ng/kg; BaP: 35–270 ng/kg; BP: 13–39 ng/kg	[17]
Milk	Saponification + LLE + SPE	HPLC–FLD	LOD: 5–110 ng/kg RSD <9% R: 65–89%	14 PAHs	Ap: 210 ng/kg; Flu: 1690 ng/kg; Phe: 720 ng/kg; Ant: 17,420 ng/kg; F: 25,860 ng/kg; Pyr: 250 ng/kg; BaA: 1280 ng/kg; Chry: 770 ng/kg; BbF: 520 ng/kg; BkF: 2450 ng/kg; BaP: 540 ng/kg; DBahA: 460 ng/kg; BP:270 ng/kg; IP: 240 ng/kg	[18]
Milk and milk powder	LLE + SPE	GC–MS	LOD: 40–75 ng/kg RSD: 3.2–10.1% R: 86–100%	16 PAHs	Nap: 20–40 ng/kg; Ap: 20–70 ng/kg; Ac: 20–30 ng/kg; F: 20–80 ng/kg; Phe: 20–110 ng/kg; Ant: 20–90 ng/kg; Flu: 30–250 ng/kg; Pyr: 120–500 ng/kg; BaA: 30–110 ng/kg; Chry: 70–300 ng/kg; BbF: 200–520 ng/kg; BkF: 20–50 ng/kg; BaP: 20–40 ng/kg; IP: 20–70 ng/kg; DBahA: 20–40 ng/kg; BP: 80–350 ng/kg	[19]
Skim milk	Magnetic SPE	HPLC–FLD	LOD: 0.2–0.6 ng/L RSD:1–9% R: 429–115%	6 PAHs	nd	[20]
Milk	SMPE	GC– MS	LOD: 100–800 ng/kg R: 75–108%	6 PAHs	Phe: 4 100 ng/kg; Ant: 900 ng/kg; Flu:800 ng/kg; Pyr 200 ng/kg	[21]
Milk	IT–SPME	HPLC–FLD	LOD: 0.10–2.36 ng/L RSD< 11% R: 76–119%	10 PAHs	Flu: 0.84–1.32 ng/L; Chry: 1.51 ng/L	[22]
Milk and dairy product	DI–SPME	GC–MS	LOD: 30–1560 ng/L RSD: 4.9–19.6% R: 88–112%	16 PAHs	Fat milk: Flu: 830–1040 ng/L; Pyr: 630–1120 ng/L	[23]
Milk	IL–HF–LPME	HPLC–DAD	LOD: 140–710 ng/L RSD 1.2−3.3% R: 94–103%	3 PAHs	nd	[24]
Milk and dairy products	Soxhlet + GPC	CG–MS/MS	–	16 PHAs	∑PHA16; Milk: 147,700 ng/kg; Cheese: 76,600 ng/kg; Yogurt: 12,800ng/kg; Butter: 7800 ng/kg	[25]
Milk	QuEChERS	GC–MS/MS	LOD: 80–150 ng/kg RSD < 6% R: 63–105%	16 PHAs	Nap: 90–1180 ng/kg; Ap: 80–120 ng/kg; Ac: 60–680 ng/kg; F: 120–1620 ng/kg; Phe: 240–920 ng/kg; Ant: 510–3850 ng/kg; Flu: 100–880 ng/kg; Pyr: 80–830 ng/kg; BaA: 490–1060 ng/kg; Chry: 220–770 ng/kg; BkF: 420–800 ng/kg; BbF: 230–880 ng/kg; BaP: 370–830 ng/kg; IP:450–1 690 ng/kg; DBahA: 240–1160 ng/kg; BP: 240–950 ng/kg	[26]
Milk	Saponification + LLE	GC–FID	LOD: 50–450 ng/kg RSD < 7.8% R: 79–99%	16 PAHs	∑PHA16: 15,600–171,180 ng/kg	[27]
Smoked cheeses	Soxhlet + SPE	GC–MS	LOQ: 900–20,000 ng/kg	16 PAHS	Nap: 20,000–1,200,000 ng/kg; Ap: 2700–1,200,000 ng/kg; Ac: 1300–38,000 ng/kg; F: 6200–400,000 ng/kg; Phe: 8500–790,000 ng/kg; Ant: 1600 ng/kg; Flu: 2700–94,000 ng/kg; Pyr: 1 600 –67 000 ng/kg; BaA: 1500–9 700 ng/kg; Chry: 1600–7300 ng/kg; BbF: 970–1100 ng/kg; BkF: 1200–2300 ng/kg; BaP: 850–4500 ng/kg	[28]
Yogurt	Saponification + LLE	HPLC–FLD	RSD: 2–20% R:33–130%	13 PHAs	Yogurt whole: Ac: 1 850 ng/kg; F: 1 400g/kg; Phe: 4 700 ng/kg; Ant: 150 ng/kg; Flu: 1 000 ng/kg; Pyr: 600 ng/kg; Chry: 50 ng/kg; DBahA: 30 ng/kg; Yogurt skimmed: Ac: 650 ng/kg; F: 770 ng/kg; Phe: 2 420 ng/kg; Ant: 80 ng/kg; Flu: 630 ng/kg; Pyr: 320 ng/kg; Chry: 30 ng/kg; DBahA: 40 ng/kg	[29]
Yogurt	Saponification + LLE	HPLC–FLD	LOD: 50–70 ng/kg RSD: 5.9−16.9% R: 84–106%	4 PAHs	BaA: 90 ng/kg; Chry: 310 ng/kg; BaP: 160 ng/kg	[31]
Milk and dairy product	LLE+ SPE	GC–MS	LOD: 1–200 ng/kg RSD: 5.0–11.3% R:80–107%	16 PHAs	Nap: 260–1 900 ng/kg; Ac: 7.1–510 ng/kg; F:30–520 ng/kg; Phe: 88 ng/kg	This work

^a^ DI–SMPE: direct immersion solid-phase microextraction; GC–MS: gas chromatography coupled to single-quadrupole mass spectrometry; GC–MS/MS: gas chromatography coupled to triple-quadrupole mass spectrometry; GPC: gel permeation chromatography; HPLC–DAD: high-performance liquid chromatography–photodiode array detection; HPLC–FLD: high-performance liquid chromatography–fluorescence detection; HPLC–MS: high-performance liquid chromatography–mass spectrometry; IL–HF–LPME: ionic liquid liquid-phase micro–extraction; IT–SPME: in-tube solid-phase microextraction; LLE: liquid-liquid extraction; LOD: detection limit; LOQ: quantification limit; QuEChERS: quick, easy, cheap, effective, rugged and safe; R: recovery; SPE: solid-phase extraction; SPME: solid-phase micro-extraction; RSD relative standard deviation. ^b^ Ac: acenaphthene; Ap: acenaphthylene; Ant: anthracene; BaA: benzo(a)anthracene; BaP: benzo(a)pyrene; BbF: benzo(b)fluoranthene; BP: benzo[g.h.i]perylene; BkF: benzo(k)fluoranthene; Chry: chrysene; DBahA: dibenzo[a.h]anthracene; F: fluorine; Flu: fluoranthene; IP: indeno[1.2.3–cd]perylene; Nap: naphthalene; Phe: phenanthrene; Pyr: pyrene; ΣPAH16: sum of the 16 PAHs; nd: not detected.

## 2. Materials and Methods

### 2.1. Chemical and Solvents

Analytical standards for the EPA’s sixteen priority PAHs (viz., Nap, acenapthylene (Ap), acenaphthene (Ac), fluorene (F), Chry, BaA, fluoranthene (Flu), Pyr, BkF, BbF, BaP, Phe, anthracene (Ant), Benzo[ghi]perylene (BP), DBahA and IP) were purchased in the highest available purity from Dr. Ehrenstofer (Augsburg, Germany), Across (Geel, Belgium) or Fluka (St. Louis, MO, USA). The internal standard (IS), triphenylphosphate, was supplied by Fluka (St. Louis, MO, USA). Reversed-phase silica with octadecyl functional groups (RP-C18) was obtained from Supelco (Madrid, Spain). Methanol (MeOH), ethanol (EtOH), ethyl acetate, *n*-hexane, acetone, acetonitrile (ACN), *N*,*N*-dimethylformamide (DMF) and 2-propanol were purchased from Merck (Darmstadt, Germany). Finally, ultrapure water was supplied by a Milli-Q system from Millipore.

All solutions were prepared individually by dissolving each analyte at 5 g/L in acetone. The stock solutions were stored at 4 °C in the dark to avoid volatilization and photodegradation. Mixed standards containing all analytes at 1 mg/L in acetone were prepared by appropriate dilution on a daily basis [3]. The eluent used was 2-propanol containing 100 μg/L IS, also prepared daily.

### 2.2. Dairy Samples

Eleven samples of different milk brands were purchased, including whole (3.6% fat content), semi-skimmed (1.6%) and skimmed (0.3%) cow’s, goat’s (3.9%) and sheep’s (6.5%) milk. On the other hand, the seventeen samples of dairy products purchased included yogurt (2.6–5.4% fat content), milkshakes (1.0–1.5%), cream (18%), custard (2.4–3.9%), cheese (12.1–13.1%), butter (81–82%) and margarine (60%). All samples were bought in Spanish supermarkets and stored in the dark at 4 °C until analysis. Each sample was analyzed in triplicate.

### 2.3. Equipemnts

Gas chromatography–mass spectrometry analyses were performed on a Focus gas chromatograph (Thermo Electron SA, Madrid, Spain) coupled to a DSQ II quadrupole mass spectrometer using an electronic ionization source (EI) and an AL/AS 3000 AutoSampler. The chromatograph was equipped with an HP-5MS capillary column (30 m, 0.25 mm i.d., 0.25 µm film thickness) from J&W (Folson, CA, USA). Helium (99.999% pure) at a constant flow rate of 1.0 mL/min was used as the carrier gas. The oven temperature was initially set at 70 °C, which was held for 2 min and followed by a 10 °C/min ramp to 240 °C and another at 15 °C/min to 290 °C, the final temperature being held for 12 min. The temperature of the transfer line was set at 280 °C, the ion source, which was operated in the EI mode (70 eV), was set at 200 °C and solvent delay was set at 5 min. The injector was used in splitless mode at 300 °C with an injected volume of 1 µL. Detection was performed in selected ion monitoring mode (SIM) for at least three characteristic ions for each analyte. The *m/z* values for each target compound are listed in Table 2.

A Centrofriger BL-II centrifuge from JP Selecta (Barcelona, Spain) was used. The continuous SPE system consisted of a peristaltic pump (Gilson, Villiers-le-Bel, France) and two Rheodyne 5041 injection valves (Cotati, CA, USA). The tubes were made of poly(vinyl chloride), and PTFE columns were custom-packed with various sorbents. All columns were conditioned by passing 1 mL of acetonitrile, 1 mL of methanol and 10 mL of ultrapure water in this sequence.

### 2.4. Extraction of PHAs

**Figure 1:**
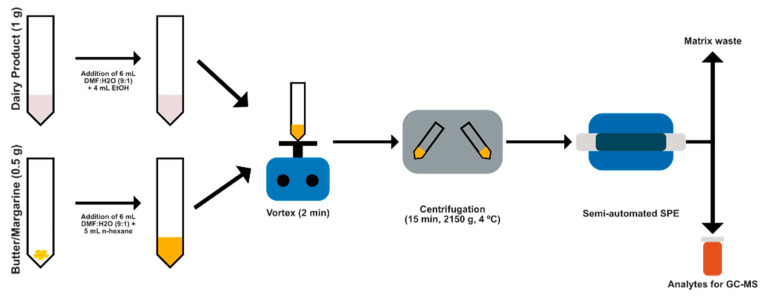
Procedure for determining polycyclic aromatic hydrocarbons in milk and dairy products.

Figure 1 depicts the extraction/cleanup procedure. Samples were defrosted—and crushed, if solid—in 50 mL polypropylene centrifuge tubes. Next, 1 g of each of the milk, yogurt, milkshake, cream, custard or cheese samples were placed into the 50 mL polypropylene centrifuge tubes and then 6 mL of a (9:1 *v/v*) DMF:H_2_O solution was added for the liquid–liquid extraction procedure. Then, 4 mL of ethanol was added, and the tube was vortexed for 2 min. By contrast, the butter and margarine samples were prepared as follows: First, 0.5 g was weighed into a 50 mL polypropylene centrifuge tube, then 6 mL of (9:1 *v/v*) DMF:H_2_O solution was added for LLE and then 5 mL of *n*-hexane was added for vortexing (2 min). Next, all milk and dairy samples were centrifuged at 2150× *g* (5000 rpm) at 4 °C for 15 min to precipitate the unsaponifiable fats and proteins from the organic layer. This resulted in two separated phases, the extractant was then collected in a new tube.

The resulting aqueous extracts were diluted to 50 mL with ultrapure water and passed at a constant flow rate of 5.0 mL/min through the continuous SPE system, equipped with a sorbent column packed with 60 mg of RP-C18 placed in the loop of the injection valve (IV_1_), (Figure S1, Supporting Information). As a result, the analytes were retained by the sorbent, and the matrix was discharged to waste. Then, the column was dried with an air stream at 5.0 mL/min in both directions for 2 min, and the other valve (IV_2_) was switched to elute the analytes with 350 µL of a 2-propanol solution containing 100 µg/L triphenylphosphate (IS) that was held in the loop of IV_2_. Finally, the eluate was collected in a 0.5 mL amber glass vial that was sealed and stored refrigerated at −18 °C until analysis by GC–MS.

### 2.5. Method Validation

The analytical performance of the proposed LLE–SPE/GC–MS method was evaluated in terms of linear range, sensitivity, selectivity, precision and accuracy. Linearity was assessed in uncontaminated samples of whole cow’s milk and butter that were spiked with variable concentrations of PAHs.

The sensitivity of the proposed method was evaluated in terms of the limits of detection (LODs), which were calculated as the signal-to-noise ratios for 3 selected ions, and ranged from 1 to 100 ng/kg for milk and from 2 to 200 ng/kg for butter. The limits of quantification (LOQs) were calculated as 3.3 times the corresponding LODs and taken to be the lower limits of the linear ranges.

Precision was evaluated as the relative standard deviation (RSD) for 12 individual samples spiked with 500, 1000 and 2000 ng/kg (milk, yogurt, cheese, custard, cream and milkshakes) or 1000, 1500 and 3000 ng/kg (butter and margarine) on the same day (within-day RSD) or 3 consecutive days (between-day RSD). Finally, the analyte recoveries were evaluated in samples spiked with three different concentrations of PAHs (500, 1000 and 2000 ng/kg (milk, yogurt, cheese, custard, cream and milkshakes) or 1000, 1500 and 3000 ng/kg (butter and margarine) for analysis in triplicate (*n* = 3).

## 3. Results and Discussion

### 3.1. Optimization of the Sample Treatment

The high complexity of milk and dairy products requires careful optimization of each variable influencing analytical performance. Milk and dairy products contain variable proportions of fat (0.3–82%) and protein (0.6–24%) that can be removed in different ways to avoid matrix effects and to isolate the analytes [33]. Often, the procedure of choice involves a saponification pretreatment in combination with LLE and followed by SPE [11,12,16,17]. In this work, we initially used saponification with NaOH in methanol in combination with LLE and SPE. The results, however, were poor. Recently, our research group succeeded in determining the PAHs in edible oils using a (9:1 *v/v*) DMF:H_2_O mixture as the extractant [3]. This led us to use a 10 mL volume of the previous mixture to extract PAHs from dairy products (a 1 g sample was spiked with a 1000 ng/kg concentration of each analyte). Although the results were somewhat better than with saponification, the PAH recoveries were far from quantitative. Tests with different extraction mixtures containing 6 mL of DMF–H_2_O solution and 4 mL of EtOH, MeOH or *n*-hexane revealed that the ethanolic mixture provided recoveries 5 times higher than the others, possibly because ethanol denatured proteins and caused them to precipitate, thereby facilitating the extraction of PAHs.

**Figure 2:**
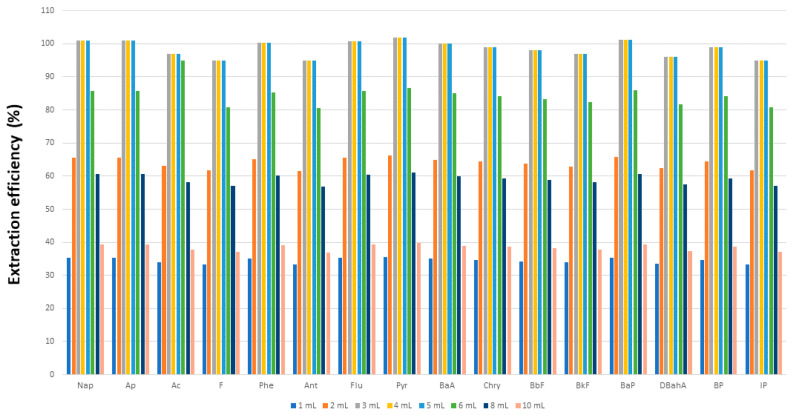
Influence of the volume of ethanol on the extraction of polycyclic aromatic hydrocarbons from dairy products.

**Figure 3:**
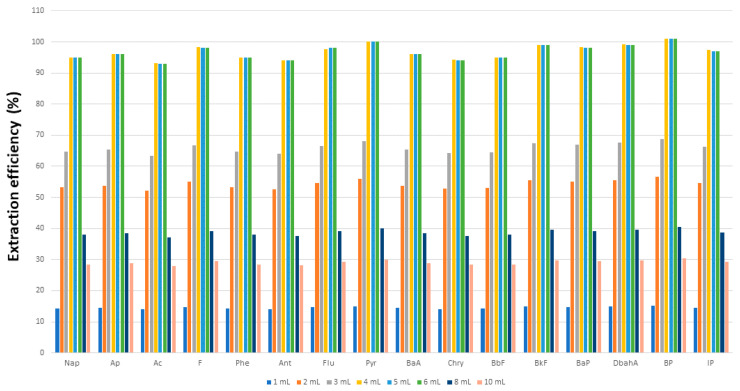
Influence of the volume of *n*-hexane on the extraction of polycyclic aromatic hydrocarbons from butter and margarine.

Extraction efficiency is typically related to the volume of organic solvent used. In this work, we established the optimum volume by spiking milk, yogurt, milkshake, cream, custard and cheese samples with a fixed analyte concentration (500 ng PAH/kg) for extraction with variable volumes (1–10 mL) of (9:1 *v/v*) DMF:H_2_O mixed with a fixed volume (4 mL) of EtOH for treatment as described in Section 2.3. The extraction yield increased with increasing volumes of DMF:H_2_O up to 6 mL, above which it levelled off. The influence of the ethanol volume was examined by using a fixed volume (6 mL) of (9:1 *v/v*) DMF:H_2_O and variable volumes of alcohol (1–10 mL). As can be seen in Figure 2, the PAH extraction efficiency peaked at EtOH volumes from 3 to 5 mL. However, using too much ethanol compromised the isolation and dilution of PAHs in water with DFM:H_2_O as the extractant [34], whereas using too little detracted from protein precipitation, so a trade-off must be made. The optimum combination for a 1 g sample of the milk and dairy products—butter and margarine excepted—was found to be 6 mL of (9:1 *v/v*) DMF:H_2_O and 4 mL of EtOH. Because butter and margarine both contained greater amounts of fat, they had to be used in smaller amounts (0.5 g instead of 1 g), spiked with a 1500 ng/kg PAH concentration and treated with 6 mL of (9:1 *v/v*) DMF:H_2_O in combination with 5 mL of solvent (methanol, ethanol or *n*-hexane) as described in Section 2.3. The best results were obtained with DMF:H_2_O–*n*-hexane, which provided near-quantitative PAH recoveries. On the other hand, methanol and ethanol provided highly cloudy extracts after centrifugation and extraction yields as low as 30% as a result. The influence of the proportion of *n*-hexane on the PAH extraction efficiency was examined by using volumes of 1–10 mL. As can be seen from Figure 3, the efficiency peaked at 4–6 mL and volumes above 6 mL decreased efficiency as a result of *n*-hexane interacting preferentially with non-polar PAHs and altering the coefficient of partition in the DMF:H_2_O mixture. We, thus, chose to use a mixture containing 6 mL of (9:1 *v/v*) DMF:H_2_O and 5 mL of *n*-hexane to extract PAHs from 0.5 g samples of margarine and butter.

The influence of centrifugation-related variables (time, temperature and speed) was examined using 1 g amounts for all milk and dairy products except butter and margarine (0.5 g) and following the PAH extraction procedure described in the previous paragraph. The temperature, centrifugation speed and time ranges studied were 0–10 °C, 430–2150 g and 1–30 min, and the optimum obtained values were 4 °C, 2150× *g* (5000 rpm) and 15 min, respectively.

Performance in the SPE step was crucial, with to the goal of ensuring that the analytes would be fully isolated from their matrices with little or no interference. The SPE–LLE combination is widely used to extract analytes [10,11,12,16,17,27]. In this work, LLE extracts were cleaned up and preconcentrated by SPE. Various sorbents, including reversed-phase silica with octadecyl groups (RP-C18), Amberlites (XAD-2 anXAD-4), Oasis HLB and LiChrolut EN, were preliminarily used to remove interferents in the aqueous layer from LLE and preconcentrate PAHs. For this purpose, the aqueous layer from the preparation of milk and dairy products was passed through an SPE column (Figure S1, Supplementary Information) packed with 60 mg of sorbent. The best results were obtained with RP-C18. Then, acetonitrile, methanol, ethanol, acetone, ethyl acetate and 2-propanol were tested as eluents and 2-propanol was found to be the most efficient in eluting retained PAHs from the sorbent column. The influence of the volume needed for complete elution of the PAHs was examined over the range of 50–500 µL, using different loops placed in the second injection valve (IV_2_ in Figure S1, Supplementary Information). The desorption efficiency increased with increasing injected volumes up to 350 µL and then decreased because of the dilution of desorbed analytes. An injection volume of eluent of 350 µL was, thus, selected as optimal.

As seen in previous studies, PAH sorption onto RP-C18 can be altered by the presence of ethanol [35]. This led us to examine the effect by passing 50 mL volumes of aqueous samples containing variable proportions of ethanol (5–50% *v/v*) and a 100 ng/L concentration of each PAH through the SPE column. The analyte elution was maximal at ethanol proportions below 20%, so we used 8% in the aqueous samples so as not to exceed that limit and detract from SPE efficiency as a result. Finally, the influence of the breakthrough volume of the RP-C18 sorbent column was examined by using aqueous solutions containing 6 mL of (9:1 *v/v*) DMF:H_2_O and 4 mL of ethanol plus a fixed amount of PAHs (5 ng) in 50–500 mL. A sorption efficiency of ca. 100% was obtained with aqueous volumes up to 250 mL.

### 3.2. Analytical Performance

**Figure 4:**
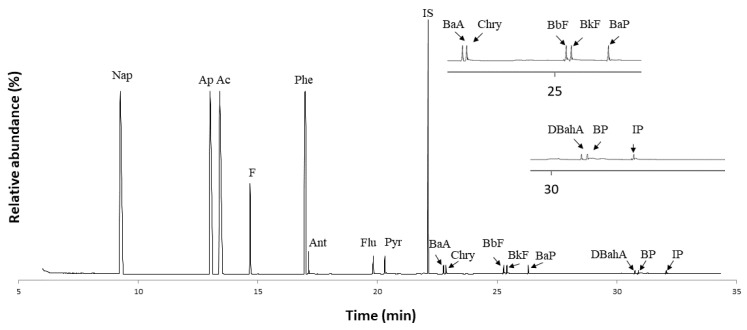
GC–MS chromatograms in the SIM mode for 1 g of whole cow’s milk spiked with a 500 ng/kg concentration of each PAH. For peak assignments, see Table 2. IS, internal standard.

With milk samples (1 g), the response of the method was linear over the range 4–20,000 ng/kg and the correlation coefficients (*r*^2^) were always higher than 0.993. For butter (0.5 g), the linear range was 7–40,000 ng/kg and the correlation coefficients were similar to those for the milk samples. Table 2 and Table S1 (Supplementary Information) list the analytical figures of merit for all types of samples and, by way of example, Figure 4 shows a typical chromatogram for a whole cow’s milk sample used to contrast the calibration curves.

Within-day RSDs were 5.0–10.2% and between-day RSDs were 6.9–11.3% (Table 2). Matrix effects (MEs) were evaluated by comparing the slopes of matrix-matched calibration curves with those of external standard calibration curves using the following equation: ME = ((slope of matrix-matched curve/slope of in-solvent curve) − 1) × 100 [36]. As can be seen from Table 3, MEs ranged from 1 to 20% and were, thus, “soft” (i.e., the sample extraction/cleanup procedure was efficient enough to avoid matrix effects on the determination of PAHs). The recoveries ranged from 80 to 107% (Table 3).

### 3.3. Analysis of Real Samples

The proposed method was used to determine the EPA’s 16 priority PAHs in 11 milk samples (viz., whole, semi-skimmed and skimmed cow’s milk, whole goat’s milk and whole sheep’s milk) and 19 dairy products (yogurt, cream, custard, cheese, milkshakes, butter and margarine). Each sample was analyzed in triplicate with an intervening blank. In each run, 1 g of sample (0.5 g for butter and margarine) was subjected to the procedure described in Section 2.4. As can be seen in Table 4, three samples (whole cow’s milk, butter and margarine) contained none of the PAHs at levels above the limit of quantification of the proposed method; however, all others contained at least one, which testifies to the ubiquity of PAHs in industrial food products.

As can be also seen in Table 4, Nap was the most frequent PAH, at concentrations from 260 to 1900 ng/kg and, especially, in margarine, butter, custard and yogurt. Chung et al. (2010) studied the presence of sixteen PAHs in milk samples from Taiwan and found Nap in all samples, at concentrations from 67,000 to 310,000 ng/L [10]. They detected additional contaminants, such as Ap, Ac, F, Flu and Phe, albeit at low concentrations. More recently, Pluta-Kubica et al. (2020) found smoked cheeses to contain 16 PAHs, with Nap as the most concentrated (20,000–1,200,000 ng/kg) [28]. By contrast, Shariarifar et al. (2020) found this PAH at much lower levels (20–40 ng/kg) in milk and milk powder [19].

Acenaphthene was present in 10 samples, at concentrations from 7.1 ng/kg in semi-skimmed milk to 510 ng/kg in butter. Two of the butter samples contained higher Ac concentrations than all others. This PAH was also found in yogurt, at levels from 650 to 1850 ng/kg, and especially in yogurt with a high fat content (3.9%) [29]. Gul et al. (2015) previously found Ac at high concentrations (110–3060 ng/kg) in cheese from Turkey [16].

Fluorene was detected in 5 samples, at 30 ng/kg in sheep’s milk and levels from 44 to 150 ng/kg in two types of cheese. One of the margarine samples contained as much as 520 ng/kg of this PAH. Sun et al. (2020) analyzed milk from 9 different countries (China, Ireland, Australia, Poland, Germany, France, Holland, Slovenia and New Zealand) and found them to contain F at concentrations from 100 to 880 ng/kg, and at especially high levels in some samples from China [26]. Fasano et al. (2016) studied cheeses from Italy, Czech Republic, Poland and Spain and found them to contain F at levels from 17,000 to 77,000 ng/kg [12].

Finally, phenanthrene was only detected, at 88 ng/kg, in one cheese sample here. Other light PAHs were also detected in some samples but never at levels above the LOQs of the proposed method. As can be seen from Table 4, the PAH concentrations found were never exceedingly high. In fact, the combined concentration of PAHs ranged from 47 ng/kg in the least contaminated sample (whole sheep’s milk) to 2120 ng/kg in one of margarine. Moreover, none of the samples contained BaP, which is one of the most hazardous PAHs according to the U.S. EPA and the EU’s Commission [13].

## 4. Conclusions

A total of sixteen PAHs deemed to be priority pollutants by the EPA were determined in milk and dairy products using an LLE–SPE/GC–MS method for the extraction, cleanup, detection and quantification of the analytes free from interferences from the sample matrix. The precision, linearity, recoveries and limits of detection afforded by the method make it an effective choice for determining the target analytes in milk and dairy samples. In fact, the method is highly sensitive, with limits of detection of 1–200 ng/kg and, thus, similar to those obtained by Lee et al. (2015) using greater amounts of sample (10 g) [11] but somewhat less so for some analytes than the method of Shariatifar et al. (2020), whish used 5 g of sample and is only applicable to milk and milk powder (Table 1) [19].

The proposed method was successfully used to analyze a wide variety of milk and dairy products, including whole, semi-skimmed and skimmed cow’s milk, goat’s milk, sheep’s milk, yogurt, milkshakes, cream, custard, cheese, butter and margarine, for PAHs. Only three samples contained no PAH at concentrations above the limit of quantitation of the method. All others contained at least one but none contained more than four. Moreover, all detected PAHs were of the lighter type (Nap, Ap, F and Phe). In any case, none of the four PAHs whose maximum levels in infant formula and follow-on milk are limited by Regulation (EU) No 835/2011 (BaP, BaA, BbF and Chry) were detected in any sample [13]. These findings offer an efficient and timely tool for evaluating the public risk associated with the presence of PAHs in milk and dairy products.

## Figures and Tables

**Table 2:** Analytical characteristics of the determination of PAHs in milk and dairy products by the proposed method.

Compound			Milk Sample							Butter Sample								^c^ *m/z*		
			^a^ LOD (ng/kg)		^b^ r		Linear Range (ng/kg)			^a^ LOD (ng/kg)		^b^ r		Linear Range (ng/kg)		t_R_		[M^+^]	Additional Ions	
Naphthalene (Nap)			1		0.9932		4–20,000			2		0.9991		7–40,000		9.22		**128**	102, 126	
Acenaphthylene (Ap)			1		0.9955		4–20,000			2		0.9942		7–40,000		12.99		**152**	151, 153, 154	
Acenaphthene (Ac)			1		0.9943		4–20,000			2		0.9956		7–40,000		13.43		**154**	152, 153	
Fluorene (F)			2		0.9941		7–20,000			5		0.9981		16–40,000		14.67		**166**	165, 167	
Phenanthrene (Phe)			1		0.9955		4–20,000			2		0.9935		7–40,000		16.98		**178**	176, 179	
Anthracene (Ant)			8		0.9956		26–20,000			17		0.9967		55–40,000		17.10		**178**	159, 176	
Fluoranthene (Flu)			10		0.9951		35–20,000			19		0.9998		60–40,000		19.82		**202**	201, 203	
Pyrene (Pyr)			10		0.9954		35–20,000			20		0.9961		65–40,000		20.32		**202**	200, 203	
Benzo(a)anthrancene (BaA)			25		0.9982		85–20,000			48		0.9989		160–40,000		22.70		**228**	226, 229	
Chrysene (Chry)			25		0.9979		85–20,000			50		0.9936		170–40,000		22.87		**228**	114, 226	
Benzo(b)fluoranthene (BbF)			25		0.9957		85–20,000			50		0.9945		170–40,000		25.31		**252**	125, 249, 250	
Benzo(k)fluoranthene (BkF)			25		0.9951		85–20,000			49		0.9971		160–40,000		25.49		**252**	249, 250	
Benzo(a)pyrene (BaP)			25		0.9966		85–20,000			50		0.9950		175–40,000		26.34		**252**	129, 253	
Dibenzo[a.h]anthracene (DBahA)			100		0.9938		350–20,000			190		0.9949		620–40,000		30.88		**278**	139, 279	
Benzo[ghi]perylene (BP)			100		0.9984		350–20,000			200		0.9935		640–40,000		31.30		**276**	272, 277	
Indeno[1.2.3–cd]pyrene (IP)			100		0.9955		350–20,000			190		0.9973		630–40,000		32.23		**276**	138, 277	
^d^ RSD (%)																				
Compound	Milk			Yogurt		Butter			Cheese				Custard		Cream		Milkshake		Margarine	
	WD	BD		WD	BD	WD		BD	WD		BD		WD	BD	WD	BD	WD	BD	WD	BD
Nap	8.6	10.5		8.5	10.3	8.2		9.8	7.0		9.0		8.7	10.1	7.9	9.3	9.0	10.1	7.5	9.2
Ap	7.6	9.3		7.9	9.4	9.8		11.0	8.7		9.4		8.6	9.6	9.2	8.4	10.0	10.5	8.0	9.5
Ac	8.9	10.5		9.5	10.9	7.9		10.8	8.6		10.5		9.1	10.5	5.0	8.9	8.7	9.3	8.1	10.0
F	8.9	9.8		8.9	11.0	8.9		10.2	5.9		9.3		7.9	8.8	7.1	10.6	8.9	10.0	6.1	8.8
Phe	7.9	10.6		9.6	11.3	8.9		10.7	9.1		10.0		5.9	7.9	7.5	9.9	8.0	11.3	9.0	10.1
Ant	8.1	9.4		9.0	10.9	7.9		10.7	10.1		11.3		8.6	10.2	8.5	10.1	7.9	9.6	8.5	11.3
Flu	8.0	8.8		9.1	10.0	5.9		8.8	8.1		9.6		7.9	11.0	9.1	10.9	8.9	9.8	8.6	9.7
Pyr	9.2	11.0		10.0	11.3	6.2		8.5	8.9		9.4		8.9	11.1	8.2	10.1	7.5	9.9	7.5	9.1
BaA	5.0	6.9		8.7	9.7	8.7		10.3	7.4		9.8		5.1	8.9	10.1	10.9	9.5	10.5	7.6	10.2
Chry	7.1	8.8		8.9	11.2	8.6		10.8	8.9		10.3		5.9	10.6	7.9	8.1	8.9	9.3	9.1	10.4
BbF	7.5	8.9		7.9	10.3	7.9		11.5	8.3		9.3		7.1	9.9	8.9	9.9	9.6	10.5	8.5	9.6
BkF	8.5	10.6		8.6	9.7	8.9		10.1	8.5		10.9		8.9	10.1	9.8	10.9	9.5	9.8	8.0	9.8
BaP	9.1	9.9		8.8	11.0	8.4		10.6	7.9		9.9		6.5	9.5	10.2	10.5	7.0	10.6	6.3	9.6
DBahA	8.9	10.1		8.9	11.2	9.0		10.1	8.6		9.2		6.6	9.9	9.9	10.7	8.7	9.5	7.2	9.0
BP	8.6	9.6		9.6	10.4	7.6		8.9	7.9		10.2		6.7	7.9	8.7	10.6	8.9	9.8	7.4	9.8
IP	7.9	8.3		7.9	10.7	8.6		10.9	7.8		10.3		7.8	8.7	9.9	10.3	5.9	8.9	8.6	10.1

^a^ LOD: detection limit; ^b^ r: correlation coefficient; t_R_: retention time; ^c^ *m/z* mass/charge ratio; [M^+^]: ionized mass; the base peaks used for quantification are boldfaced; *m/z* for IS (triphenylphosphate): 170, 325, **326**; ^d^ RSD. relative standard deviation (*n* = 12), values obtained for samples fortified with 500 ng/kg, except for butter and margarine, which was 1000 ng/kg; WD: within-day; BD: between-day.

**Table 3:** Results obtained in the recovery and matrix effect studies of the different types of milk and dairy product samples.

	**Recoveries (% ± SD. *n* = 3) ^a^**							
	**Milk**	**Yogurt**	**Butter**	**Cheese**	**Custard**	**Cream**	**Milkshake**	**Margarine**
Naphthalene	101 ± 8	100 ± 9	95 ± 8	99 ± 9	98 ± 9	99 ± 9	100 ± 10	96 ± 9
Acenaphthylene	101 ± 8	105 ± 8	92 ± 8	107 ± 10	99 ± 9	97 ± 8	97 ± 10	105 ± 9
Acenaphthene	82 ± 8	106 ± 10	93 ± 10	104 ± 10	89 ± 8	93 ± 8	100 ± 9	106 ± 10
Fluorene	91 ± 6	107 ± 10	98 ± 10	97 ± 9	101 ± 8	105 ± 10	91 ± 2	107 ± 9
Phenanthrene	100 ± 9	104 ± 9	93 ± 9	101 ± 10	89 ± 7	99 ± 10	95 ± 9	104 ± 10
Anthracene	95 ± 8	101 ± 9	88 ± 8	96 ± 10	105 ± 10	87 ± 8	102 ± 11	101 ± 11
Fluoranthene	101 ± 3	94 ± 8	98 ± 8	96 ± 9	100 ± 11	93 ± 9	97 ± 10	94 ± 9
Pyrene	102 ± 10	94 ± 8	104 ± 9	87 ± 8	99 ± 10	100 ± 11	105 ± 10	95 ± 9
Benzo(a)anthracene	106 ± 10	97 ± 9	85 ± 8	86 ± 8	105 ± 9	89 ± 8	99 ± 11	106 ±10
Chrysene	105 ± 8	82 ± 6	94 ± 10	87 ± 8	99 ± 10	102 ± 6	101 ± 9	82 ± 8
Benzo(b)fluoranthene	94 ± 6	106 ± 9	95 ± 10	83 ± 7	100 ± 10	97 ± 8	98 ± 10	106 ± 10
Benzo(k)fluoranthene	97 ± 9	93 ± 8	104 ± 10	102 ± 9	105 ± 5	96 ± 9	103 ± 10	93 ± 9
Benzo(a)pyrene	101 ± 9	85 ± 6	98 ± 10	83 ± 8	99 ± 9	106 ± 10	89 ± 8	85 ± 8
Dibenzo[a,h]anthracene	87 ± 8	93 ± 8	99 ± 10	86 ± 7	102 ± 10	105 ± 11	99 ± 10	93 ± 8
Benzo[g,h,i]perylene	99 ± 9	90 ± 7	106 ± 9	86 ± 7	103 ± 8	99 ± 10	94 ± 9	90 ± 9
Indeno[1,2,3-cd]pyrene	80 ± 6	100 ± 10	97 ± 9	95 ± 9	99 ± 9	100 ± 10	101 ± 9	100 ± 10
**Matrix Effect (%) ^b^**								
	**Milk**	**Yogurt**	**Butter**	**Cheese**	**Custard**	**Cream**	**Milkshakes**	**Margarine**
Naphthalene	1.02 (2%)	0.86 (−14%)	0.92 (−8%)	0.99 (−1%)	0.91 (−9%)	0.91 (−9%)	1.02 (2%)	1.03 (3%)
Acenaphthylene	1.10 (10%)	0.96 (−4%)	0.94 (−6%)	1.03 (3%)	0.93 (−7%)	0.99 (−1%)	0.96 (−4%)	1.06 (6%)
Acenaphthene	1.09 (9%)	0.91 (−9%)	1.03 (3%)	1.06 (6%)	0.85 (−15%)	0.86 (−14%)	1.06 (6%)	0.93 (−7%)
Fluorene	1.10 (10%)	0.99 (−1%)	0.85 (−15%)	0.90 (−10%)	1.06 (6%)	1.06 (6%)	0.99 (−1%)	1.03 (3%)
Phenanthrene	1.03 (3%)	1.03 (3%)	0.88 (−12%)	0.92 (−8%)	1.06 (6%)	0.93 (−7%)	0.90 (−10%)	1.12 (12%)
Anthracene	1.06 (6%)	1.06 (6%)	0.90 (−10%)	0.81 (−19%)	1.03 (3%)	0.90 (−10%)	1.03 (3%)	0.90 (−10%)
Fluoranthene	0.91 (−9%)	0.93 (−7%)	0.84 (−16%)	0.90 (−10%)	1.03 (3%)	0.97 (−3%)	0.99 (−1%)	1.10 (10%)
Pyrene	0.97 (−3%)	1.14 (14%)	1.14 (14%)	0.82 (−18%)	0.97 (−3%)	1.03 (3%)	1.03 (3%)	0.99 (−1%)
Benzo(a)anthracene	1.03 (3%)	1.12 (12%)	1.04 (4%)	0.82 (−18%)	0.90 (−10%)	0.93 (−7%)	0.92 (−8%)	1.06 (6%)
Chrysene	1.03 (3%)	0.95 (−5%)	0.96 (−4%)	0.83 (−17%)	1.03 (3%)	1.07 (7%)	1.06 (6%)	0.93 (−7%)
Benzo(b)fluoranthene	1.05 (5%)	1.02 (2%)	0.81 (−19%)	0.82 (−18%)	1.14 (14%)	0.91 (−9%)	0.93 (−7%)	0.88 (−12%)
Benzo(k)fluoranthene	1.03 (3%)	1.04 (4%)	0.98 (−2%)	0.84 (−16%)	1.04 (4%)	0.99 (−1%)	1.03 (3%)	1.04 (4%)
Benzo(a)pyrene	1.09 (9%)	0.93 (−7%)	0.94 (−6%)	0.90 (−10%)	0.93 (−7%)	1.03 (3%)	0.82 (−18%)	1.06 (6%)
Dibenzo[a,h]anthracene	1.06 (6%)	1.07 (7%)	0.93 (−7%)	0.82 (−18%)	1.03 (3%)	1.06 (6%)	0.83 (−17%)	1.14 (14%)
Benzo[g,h,i]perylene	1.05 (5%)	1.16 (16%)	1.03 (3%)	1.06 (6%)	1.05 (5%)	0.97 (−3%)	0.82 (−18%)	0.93 (−7%)
Indeno[1,2,3-cd]pyrene	0.86 (−14%)	1.19 (19%)	0.86 (−14%)	1.06 (6%)	0.99 (−1%)	1.06 (6%)	1.02 (2%)	1.06 (6%)

^a^ Percent recoveries (% ± SD, *n* = 3) of PAHs spiked to milk and dairy product samples (500 ng/kg, except for butter and margarine, which were 1000 ng/kg). ^b^ Matrix effects are expressed as the ratio between the calibration curve slope in matrix and the calibration curve slope in solvent. The result of the following operation is included in parentheses: ((calibration curve slope in matrix/calibration curve slope in solvent) − 1) × 100.

**Table 4:** Polycyclic aromatic hydrocarbons (mean values ± standard deviation, ng/kg) found in various types of milk samples and dairy products (*n* = 3).

Sample ^a^	Compounds	Concentration Found (ng/kg)	Sample ^a^	Compounds	Concentration Found (ng/kg)	Sample ^a^	Compounds	Concentration Found (ng/kg)
Skimmed cow’s milk 1 (0.3%/3.3%)	Naphthalene Acenaphthene ΣPAH	580 ± 50 50 ± 5 630 ± 50	Whole sheep´s milk (6.5%/5.4%)	Acenaphthene Fluorene ΣPAH	17 ± 2 30 ± 3 47 ± 4	Custard 3 (3.9%/3.4%)	Naphthalene	1400 ± 100
Skimmed cow’s milk 2 (0.3%/3.3%)	Naphthalene	620 ± 60	Yoghurt cow´s 1 (2.6%/3.9%)	Naphthalene Acenaphthene ΣPAH	860 ± 80 51 ± 5 911 ± 80	Cheese 1 (12.1%/10.9%)	Naphthalene	490 ± 40
Skimmed cow´s milk 3 (0.3%/3.3%)	Naphthalene	800 ± 70	Yoghurt cow´s 2 (3.0%/3.5%)	Naphthalene Acenaphthene ΣPAH	1500 ± 100 35 ± 3 1535 ± 100	Cheese 2 (13.1%/12.9%)	Naphthalene Acenaphthene Fluorene Phenanthrene ΣPAH	900 ± 80 50 ± 5 44 ± 4 88 ± 8 1082 ± 80
Semi-skimmed cow´s milk 1 (1.6%/4.9%)	Naphthalene Acenaphthene ΣPAH	560 ± 50 7.1 ± 0.6 567.1 ± 50	Yoghurt cow´s 3 (2.6%/3.9%)	Naphthalene Acenaphthene ΣPAH	1100 ± 100 35 ± 3 1135 ± 100	Cheese 3 (12.9%/10.1%)	Fluorene	150 ± 10
Semi-skimmed cow´s milk 2 (1.6%/4.9%)	Naphthalene	530 ± 40	Milkshake 1 (1.0%/1.6%)	Naphthalene	580 ± 50	Butter (82.0%/0.7%)	Naphthalene Acenaphthene ΣPAH	1000 ± 100 300 ± 30 1300 ± 100
Semi-skimmed cow´s milk 2 (1.6%/4.9%)	Naphthalene	570 ± 50	Milkshake 2 (1.0%/1.6%)	Naphthalene	260 ± 20	Butter (81.0%/0.5%)	Naphthalene Acenaphthene ΣPAH	440 ± 40 510 ± 50 950 ± 60
Whole cow´s milk 1 (3.6%/3.2%)	Naphthalene	380 ± 30	Cream 1 (18.0%/2.4%)	Naphthalene	330 ± 30	Butter (82.2%/0.5%)	nq ^b^	
Whole cow´s milk 2 (3.6%/3.2%)	Naphthalene	520 ± 40	Cream 2 (18.1%/2.5%)	Naphthalene	290 ± 30	Margarine (60.0%/0.5%)	Naphthalene Fluorene ΣPAH	1200 ± 100 520 ± 50 1720 ± 110
Whole cow´s milk 3 (3.6%/3.2%)	nq ^b^		Custard 1 (2.9%/3.4%)	Naphthalene	960 ± 90	Margarine (60.0%/0.5%)	Naphthalene Fluorene ΣPAH	1900 ± 200 220 ± 20 2120 ± 200
Whole goat´s milk (3.9%/3.4%)	Naphthalene Acenaphthene ΣPAH	550 ± 50 23 ± 2 573 ± 50	Custard 2 (2.4%/2.3%)	Naphthalene	780 ± 70	Margarine (60.5%/0.5%)	nq^b^	

^a^ (% fat content/ protein content). Samples of milk or dairy products are of different brands. ^b^ nq: PAHs concentration < LOQ ΣPAH includes the sum of all PAH.

## Data Availability

Not applicable.

## Supplementary Materials

The following supporting information can be downloaded at: https://www.mdpi.com/article/10.3390/foods11050713/s1, Figure S1: Continuous-flow system for the solid-phase extraction of PAHs from dairy products; Table S1: Regression equation and quantification limit of the determination of PAHs in milk and dairy product by the proposed method.
