# Supplementary material for: Trace-Level Determination of Polycyclic Aromatic Hydrocarbons in Dairy Products Available in Spanish Supermarkets by Semi-Automated Solid-Phase Extraction and Gas Chromatography–Mass Spectrometry Detection
Source: Foods. 2022 Feb 28;11(5):713. doi: 10.3390/foods11050713 (PMC8909477; doi:10.3390/foods11050713)
Supplement: Supplementary file 1 [file foods-11-00713-s001.zip › foods-1614459-supplementary.pdf]

Supplementary Information

Trace-Level Determination of Polycyclic Aromatic Hydrocarbons in Dairy Products Available in Spanish Supermarkets by Semi-Automated Solid-Phase Extraction and Gas Chromatography–Mass Spectrometry Detection

Laura Palacios Colón, Andrés J. Rascón, Evaristo Ballesteros*

Department of Physical and Analytical Chemistry, E.P.S of Linares, University of Jaén, 23700 Linares, Jaén, Spain

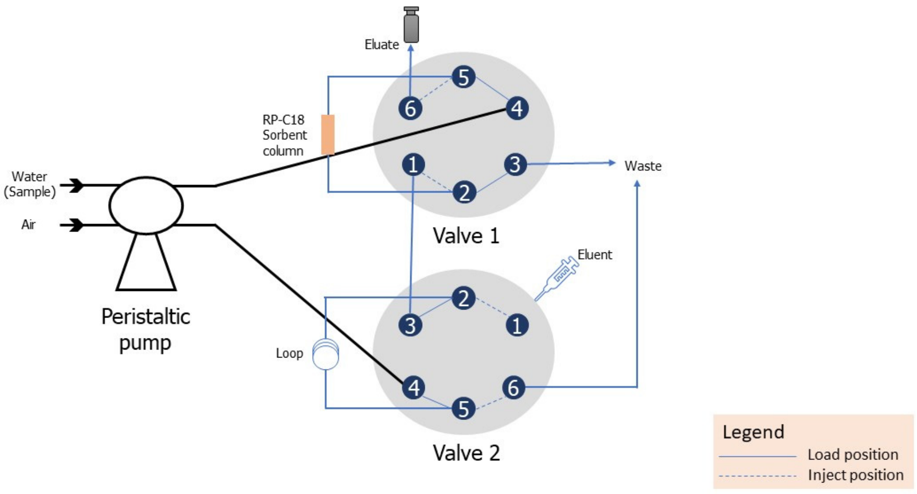

Figure 1S. Continuous-flow system for the solid-phase extraction of PAHs from dairy products.

Table 1S. Regression equation and quantification limit of the determination of PAHs in milk and dairy product by the proposed method.

Compound	Milk sample		Butter sample	
	LOQ[a] (ng/kg)	Regression equation	LOQ[a] (ng/kg)	Regression equation
Naphthalene (Nap)	4	$y= 9.3\cdot10^{-3}$ x + 0.003	7	$y= 4.2\cdot10^{-3}$ X + 0.002
Acenaphthylene (Ap)	4	$y= 8.5\cdot10^{-3}$ x + 0.006	7	$y= 5.1\cdot10^{-3}$ X - 0.004
Acenaphthene (Ac)	4	$y= 9.1\cdot10^{-3}$ x + 0.002	7	$y= 4.1\cdot10^{-3}$ X + 0.003
Fluorene (F)	7	$y= 5.2\cdot10^{-3}$ x + 0.006	16	$y= 2.4\cdot10^{-3}$ X - 0.005
Phenanthrene (Phe)	4	$y= 8.2\cdot10^{-3}$ x + 0.005	7	$y= 4.5\cdot10^{-3}$ X- 0.002
Anthracene (Ant)	26	$y= 9.4\cdot10^{-4}$ x + 0.008	55	$y= 4.6\cdot10^{-4}$ X + 0.003
Fluoranthene (Flu)	35	$y= 8.5\cdot10^{-4}$ x + 0.004	60	$y= 4.2\cdot10^{-4}$ X + 0.004
Pyrene (Pyr)	35	$y= 8.8\cdot10^{-4}$ x - 0.003	65	$y= 4.0\cdot10^{-4}$ X + 0.006
Benzo(a)anthrancene (BaA)	85	$y= 4.3\cdot10^{-4}$ x - 0.005	160	$y= 2.0\cdot10^{-4}$ X + 0.001
Chrysene (Chry)	85	$y= 4.0\cdot10^{-4}$ x + 0.007	170	$y= 1.8\cdot10^{-4}$ X + 0.002
Benzo(b)fluoranthene (BbF)	85	$y= 3.5\cdot10^{-4}$ x + 0.009	170	$y= 1.6\cdot10^{-4}$ X -0.001
Benzo(k)fluoranthene (BkF)	85	$y= 3.6\cdot10^{-4}$ x + 0.002	160	$y= 1.4\cdot10^{-4}$ X + 0.007
Benzo(a)pyrene (BaP)	85	$y= 3.0\cdot10^{-4}$ x - 0.001	175	$y= 1.5\cdot10^{-4}$ X - 0.003
Dibenzo[a.h]anthracene (DBahA)	350	$y= 0.8\cdot10^{-4}$ x - 0.004	620	$y= 0.4\cdot10^{-4}$ X - 0.002
Benzo[ghi]perylene (BP)	350	$y= 0.7\cdot10^{-4}$ x + 0.005	640	$y= 0.4\cdot10^{-4}$ X + 0.002
Indeno[1.2.3–cd]pyrene (IP)	350	$y= 0.8\cdot10^{-4}$ x - 0.002	630	$y= 0.5\cdot10^{-4}$ X - 0.003

[a] LOQ: quantification limit
